# Supplementary material for: Ocular biometric parameters changes and choroidal vascular abnormalities in patients with neurofibromatosis type 1 evaluated by OCT-A
Source: PLoS One. 2021 May 6;16(5):e0251098. doi: 10.1371/journal.pone.0251098 (PMC8101920; doi:10.1371/journal.pone.0251098)
Supplement: Attachment — Submitted filename: PLOS one Answers reviewers.docx [file pone.0251098.s001.docx]

**Reviewer #1: This is an interesting paper in an area of active research (biomarkers for NF1) that has rapidly progressed with multimodal imaging. The use of swept source and OCTA by the authors provides further knowledge on the topic.
Some revision would greatly enhance the manuscript as follows:

Abstract
Specify “macular” RNFL, if this is the case. This needs to be specified in the methods section and throughout.**
We thank the reviewer for this comment; we specified it was the RFNL thickness measured in the macular region. **In the conclusions avoid general comments (first sentence) and focus on specific results such as the major novel finding ie choroidal and retinal vascular flow area. The use of the term “diagnostic tool” may be appropriate once further studies are carried out, rather the findings of the authors provide further knowledge on the vascular alterations of the choroid using advance imaging, and thinning of the RNFL even in the absence of optic nerve gliomas**

We thank the reviewer for this insight. We avoided general comments as suggested, focusing on the possible importance of our findings and we agree with the fact that OCT-A ca not be considered a diagnostic tool yet considering the relatively small evidence in this regard to date. **In the results section please check language: the word “compared” is inserted twice**

We corrected it as suggested**.

Introduction
Although the diagnosis of NF1 is clinical, some centers use genetic testing for assessment, please mention this.**

We specified it as suggested by the reviewer **Please correct to: …contemporary presence of “the” following criteria…**

We modified it as suggested by the reviewer **Please correct to: “The presence of choroidal nodules, detected as bright patchy areas by NIR, has previously been reported in 70% of pediatric patients with NF1.”**
We modified it as suggested **Please correct to: Furthermore, in patients with NF1, the association between choroidal nodules and retinal vascular abnormalities (RVAs) has been reported.**
We modified it as suggested **Please correct: OCTA has “enabled” us**

We modified it as suggested by the reviewer. **Methods
Please state if IOP was measured and please state if patients with glaucoma or borderline IOP were excluded, as this can influence RNFL and GCL- IPL thickness values.
Please correct: Remove the word “Furthermore”…… 3 different levels (l1, L2, and L3…)**
We added the corrections suggested **Results
Please evaluate RNFL and GCL excluding patients with glioma in order to confirm significance results. If, following statistical analysis, the results change please comment accordingly in discussion.**

We thank the reviewer for this comment**.** As correctly stated by reviewer cases of gliomas were present in our cohort; however, considering the post chiasmatic/optic radiations position of the finding, we did not rule out these patients from the statistical analysis. In this regard, no previous evidence has been reported in literature about a possible decrease in vascular density by OCTA in the choroid of patients with postchiasmatic gliomas. Moreover, we deem that n=3/31 patients does not represent a significant number of patients interfering with the significance of the findings in our study. **Discussion
The authors state: “A reduced RNFL thickness was previously reported in patients with NF1 and optic pathway gliomas (17,18). Thus, the measurement of RNFL thickness has been proposed as a useful tool to screen NF1 patients for the presence of gliomas (17). However, only 3 patients in our cohort had a optic pathway glioma. Therefore, RNFL thickness may be reduced in patients with NF1 even in the absence of this complication.” There are publications on RNFL thinning in patients with optic nerve glioma (Chang L, et al. Optical coherence tomography in the evaluation of neurofibromatosis type-1 subjects with optic pathway gliomas. J AAPOS 2010; 14:511-517.; Topcu-Yilmaz P, et al. Investigation of retinal nerve fiber layer thickness in patients with neurofibromatosis-1. Jpn J Ophthalmol 2014; 88:172-176.)
but also RNFL thinning in adult patients WITHOUT optic nerve glioma (Abdolrahimzadeh S, et al. Spectral domain optical coherence tomography evidence of retinal nerve fibre layer and ganglion cell loss in adult patients with neurofibromatosis type 1. Retina 2016; 36:75-81). Furthermore, some authors have studied the peripapillary RNFL and some also the macular RNFL.

This section would benefit with some discussion, please mention the relevant references above when discussing the results on RNFL thickness.**

We thank the reviewer for this comment. We added the suggested studies dealing with the study of RNFL thickness in NF1 patients without glioma, discussing its potential role for further studies. **Citations and references
Please correct citations in text: in some sections the citation in the text is the author name and year of publication, in other sections there are numbers. Furthermore, references are not identified by numbers in the reference section. Kindly correct citations and references according to journal instructions.**

We modified the references style as suggested by the reviewer **Reference 16 is missing in the reference section when the authors refer to the previous study on choroidal thickness, Br J Ophthalmol 2015;99:789-793.

References 17, and 18 are missing in the reference section when the authors refer to RNFL thinning, Chang L et al and Topcu-Yilmaz P et al.

Please carefully check citations and reference list for accuracy throughout.**

All the references have been carefully checked and reordered

**Reviewer #2: Very interesting study
one major remark: No data on MRI of the brain was given, if not all NF1 patients and NF1 suspected patients have undergone an MRI, no conclusions related to the RNFL can be drawn**

We thank the reviewer for the comment. We deem that our study has an ophthalmological perspective and the evaluation of MRI data, beside the individuation of 3 postchiasmatic gliomas in 3 out 31 patients with confirmed diagnosis of NF1, falls outside the topic of this study. Moreover, MRI findings are not very unlikely to interfere with the alterations reported in choroidal vasculature and RNFL thinning measure by OCT.
We better discussed our findings dealing with RNFL thinning in the discussion paragraph.
